# REM sleep behaviour disorder in patients without synucleinopathy

**DOI:** 10.1136/jnnp-2020-323475

**Published:** 2020-08-13

**Authors:** Lindsay H M Keir, David P Breen

**Affiliations:** 1 Department of Clinical Neurosciences, Royal Infirmary of Edinburgh, Edinburgh, UK; 2 Centre for Clinical Brain Sciences, University of Edinburgh, Edinburgh, UK; 3 Anne Rowling Regenerative Neurology Clinic, University of Edinburgh, Edinburgh, UK; 4 Usher Institute of Population Health Sciences and Informatics, University of Edinburgh, Edinburgh, UK

**Keywords:** sleep disorders, lewy body, movement disorders, pathology, post mortem

REM sleep behaviour disorder (RBD) is a parasomnia characterised by the loss of muscle atonia in REM sleep, leading to dream enactment behaviours with or without vocalisations. ‘Idiopathic’, or perhaps more accurately isolated, RBD occurs in the absence of an underlying neurological disorder and has a prevalence of 0.38% to 1.15% in the general population aged over 60 years.^1 2^ It is recognised that RBD frequently represents an early prodromal marker of an emerging synucleinopathy (Parkinson’s disease, dementia with Lewy bodies or multiple system atrophy), with over 70% of individuals developing one of these diseases over a 12-year follow-up.^3^ However, RBD does not occur exclusively with synucleinopathies. We set out to perform a systematic review to describe the spectrum of other neurological disorders associated with RBD.

We searched PubMed on 1^st^ December 2019 to identify all previous reports of polysomnography-confirmed RBD cases occurring in association with neurological conditions (online supplementary figure). The search terms ‘rapid eye movement sleep behaviour disorder’ OR ‘rapid eye movement sleep behavior disorder’ OR ‘RBD’ were used. Only full-text articles published in the English language were considered. Pathological diagnoses were prioritised if this information was available; where mixed pathologies were reported, cases were excluded if one was a synucleinopathy. Papers were excluded if the patients had been reported previously. Cases of subclinical RBD (defined as confirmed REM sleep without atonia in the absence of RBD symptoms), cognitive impairment or neurological presentations of uncertain aetiology, or RBD occurring in healthy individuals (usually children) were excluded. We did not include RBD cases associated with primary sleep disorders such as narcolepsy (which is known to be associated with RBD) or obstructive sleep apnoea (which can co-exist with RBD or directly lead to RBD-like movements during apnoeic episodes). RBD cases occurring while taking antidepressant medications were excluded, even if the patient had another neurological condition, since it remains unknown whether this reflects a reversible side effect or an unmasking of RBD in a patient subgroup that are at increased risk of developing a defined neurodegenerative syndrome. Although RBD has been associated with psychiatric disorders, these cases were excluded since they often occurred in the context of other neurological conditions and/or antidepressants.

10.1136/jnnp-2020-323475.supp1 Supplementary data

We found 237 patients with polysomnography-confirmed RBD occurring in association with neurological conditions not caused by alpha-synuclein pathology (table 1). Structural brain lesions, typically in the brainstem, accounted for 19% of cases (n=45). While ischaemic lesions were the predominant aetiology, other lesions were also reported (eg, inflammatory, neoplastic, vascular malformations). The importance of the brainstem location, as opposed to the underlying disease mechanism, was further highlighted by co-occurrence of RBD and Arnold-Chiari malformations in 29 patients (12%).

**Table 1:** Non-synuclein pathologies associated with RBD

Condition	Number of patients	Subcategory	Supplementary References
Structural brain lesion	45	Ischaemic (n=23)*, multiple sclerosis (n=7), indeterminate lesion (n=4), meningioma (n=3), adult-onset leukodystrophy (n=2), post-operative following brain surgery (n=1), brainstem cavernoma (n=1), aneurysm (n=1), brainstem lymphoma (n=1), astrocytoma (n=1), anoxic encephalopathy (n=1)	1,2, 3†, 4†,5,6, 7‡, 8-12, 13§, 14, 15, 16¶, 17-21
Arnold-Chiari malformation	29	Type 1 (n=6), Type II (n=23)	22,23
Alzheimer’s disease	29	na	13§, 24-31, 32§
Spinocerebellar ataxia	25	SCA-3 (n=20), SCA of uncertain aetiology (n=4), SCA-31 (n=1)	30, 33-38
Autoimmune brain disease	22	Voltage-gated potassium channel antibody encephalitis (n=9), paediatric acute-onset neuropsychiatric syndrome (n=4), anti-Ma antibody encephalitis (n=3), encephalitis of uncertain aetiology (n=3), paraneoplastic cerebellar degeneration (n=2), NMDA receptor encephalitis (n=1)	6, 13§, 16¶, 39-42, 43§, 44, 45, 46
Epilepsy	19	na	15, 47-49
Progressive supranuclear palsy	15	na	6, 13§, 50, 51, 52§, 53
Traumatic brain injury	11	na	2, 3†, 54. 55
Guadeloupean parkinsonism**	7	na	51
Wilson’s disease	6	na	56, 57

This table highlights neurological conditions previously associated with polysomnography-confirmed RBD. Only conditions with five or more cases are shown, with the following conditions being observed less frequently: amyotrophic lateral sclerosis (n=4), dentatorubropallidoluysian atrophy (n=4), anti-IgLON5 disease (n=4), autism (n=4), Huntington’s disease (n=3), Creutzfeldt-Jakob disease (n=3), Mobius syndrome (n=2), Guillain-Barré syndrome (n=1), frontotemporal dementia (n=1), Tourette’s syndrome (n=1), myotonic dystrophy type 2 (n=1) and hereditary hyperekplexia (n=1).

*Mainly involving the pons.

†RBD occurred as part of parasomnia overlap syndrome.

‡Not located in the brainstem and one patient also had epilepsy.

§Pathologically confirmed cases.

¶Two patients did not undergo polysomnography but insufficient information from paper to confirm which ones.

**This is a form of tauopathy.

na, not applicable; RBD, REM sleep behaviour disorder; SCA, spinocerebellar ataxia.

While RBD is typically regarded as an early feature of synucleinopathies, some patients had other degenerative brain diseases, specifically Alzheimer’s pathology (n=29, 12%) or tauopathy (n=22, 9%). We considered whether some of these cases may have been misdiagnosed synucleinopathy cases, however several of them were pathological studies which makes this less likely. Autoimmune encephalitis was observed in 22 patients (9%), which is not surprising since these conditions are known to sometimes disrupt the sleep-wake state. Epilepsy (n=19, 8%), spinocerebellar ataxia (particularly SCA-3 (n=20, 8%)), traumatic brain injury (n=11, 5%) and Wilson’s disease (n=6, 3%) were among the other causes observed.

Given the prevalence of RBD in the general population, we acknowledge that some of the reported associations may have been a coincidence and some of the patients might have gone on to develop a synucleinopathy over time. We also accept that there may be some misclassification of conditions that were diagnosed clinically in the absence of histopathology. Furthermore, our search strategy may have underestimated the overall number of cases due to the inclusion of only polysomnography-confirmed RBD cases, as well as the number of RBD cases associated with psychiatric conditions due to exclusion of these individuals (as a result of the potentially confounding influence of antidepressant medications).

Our findings indicate that a spectrum of neurological pathologies, many affecting the brainstem, can be associated with polysomnography-confirmed RBD, and these should be considered when the history or examination is not indicative of a synucleinopathy. It seems likely that synucleinopathies lead to RBD by damaging these same brainstem regions as a result of them being particularly vulnerable to synuclein-mediated neurodegeneration. Normal REM sleep atonia is believed to be under the control of the subcoeruleus in the brainstem (which sends inhibitory projections to spinal motor neurons),^4^ with disrupted glutamate transmission in this region being particularly implicated in RBD.^5^

10.1136/jnnp-2020-323475.supp2 Supplementary data
