# Supplementary material for: REM sleep behaviour disorder in patients without synucleinopathy
Source: J Neurol Neurosurg Psychiatry. 2020 Aug 13;91(11):1239–40. doi: 10.1136/jnnp-2020-323475 (PMC7569384; doi:10.1136/jnnp-2020-323475)

Supplementary figure. Flow diagram illustrating the number of articles identified through database search and number of articles excluded at each stage

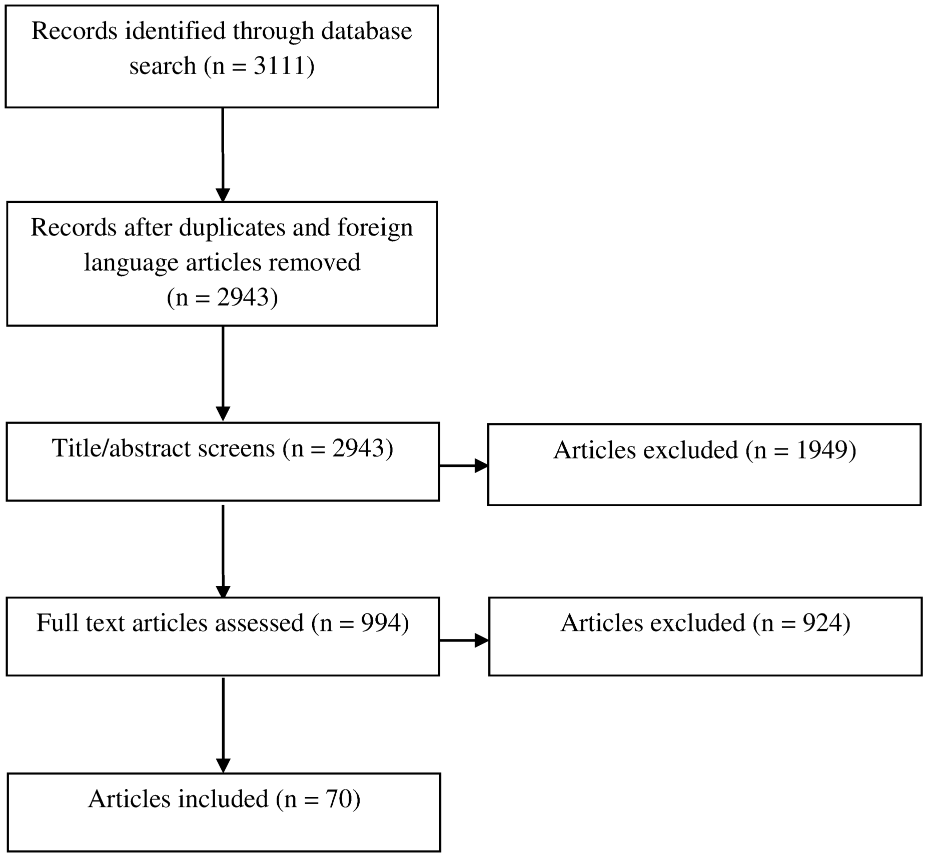

Supplement: Supplementary data [file jnnp-2020-323475supp001.pdf]
