# Supplementary material for: REM sleep behaviour disorder in patients without synucleinopathy
Source: J Neurol Neurosurg Psychiatry. 2020 Aug 13;91(11):1239–40. doi: 10.1136/jnnp-2020-323475 (PMC7569384; doi:10.1136/jnnp-2020-323475)
Supplement: Supplementary data [file jnnp-2020-323475supp002.pdf]

Supplementary references

1. Culebras A, Moore JT. Magnetic resonance findings in REM sleep behavior disorder. *Neurology* 1989; 39: 1519-1523.
2. Schenck CH, Hurwitz TD, Mahowald MW. REM sleep behaviour disorder: an update on a series of 96 patients and a review of the world literature. *J Sleep Res* 1993; 2: 224-231.
3. Schneck CH, Boyd JL, Mahowald MW. A parasomnia overlap disorder involving sleepwalking, sleep terrors, and REM sleep behavior disorder in 33 polysomnographically confirmed cases. *Sleep* 1997; 20: 972-981.
4. Limousin N, Dehais C, Gout O, Héran F, Oudiette D, Arnulf I. A brainstem inflammatory lesion causing REM sleep behavior disorder and sleepwalking (parasomnia overlap disorder). *Sleep Med* 1999; 10: 1059-1062.
5. Kimura K, Tachibana N, Kohyama J, Otsuka Y, Fukazawa S, Waki R. A discrete pontine ischemic lesion could cause REM sleep behavior disorder. *Neurology* 2000; 55: 894-895.
6. Olson EJ, Boeve BF, Silber MH. Rapid eye movement sleep behaviour disorder: demographic, clinical and laboratory findings in 93 cases. *Brain* 2000; 123: 331-339.
7. Husain AM, Miller PP, Carwile ST. REM sleep behavior disorder: potential relationship to post-traumatic stress disorder. *J Clin Neurophysiol* 2001; 18: 148-157.
8. Plazzi G, Montagna P. Remitting REM sleep behavior disorder as the initial symptom of multiple sclerosis. *Sleep Med* 2002; 3: 437-439.

9. Gomez-Choco MJ, Iranzo A, Blanco Y, Graus F, Santamaria J, Saiz A. Prevalence of restless legs syndrome and REM sleep behavior disorder in multiple sclerosis. *Multiple Sclerosis* 2007; 13: 805-808.

10. Mathis J, Hess CW, Bassetti C. Isolated mediotegmental lesion causing narcolepsy and rapid eye movement sleep behaviour disorder: a case evidencing a common pathway in narcolepsy and rapid eye movement sleep behaviour disorder. *J Neurol Neurosurg Psychiatry* 2007; 78: 427-429.

11. Xi Z, Luning W. REM sleep behavior disorder in a patient with pontine stroke. *Sleep Med* 2009; 10: 143-146.

12. Frauscher B, Gschliesser V, Brandauer E, et al. REM sleep behavior disorder in 703 sleep-disorder patients: the importance of eliciting a comprehensive sleep history. *Sleep Med* 2010; 11: 167-171.

13. Boeve BF, Silber MH, Ferman TJ, et al. Clinicopathologic correlations in 172 cases of rapid eye movement sleep behavior disorder with or without a coexisting neurologic disorder. *Sleep Med* 2013; 14: 754-762.

14. Jianhua C, Xiugin L, Quancai C, Heyang S, Yan H. Rapid eye movement sleep behavior disorder in a patient with brainstem lymphoma. *Intern Med* 2013; 52: 617-621.

15. Khalil A, Wright MA, Walker MC, Eriksson SH. Loss of rapid eye movement sleep atonia in patients with REM sleep behavioural disorder, narcolepsy, and isolated loss of REM atonia. *J Clin Sleep Med* 2013; 9: 1039-1048.

16. McCarter SJ, Tippmann-Peikert M, Sandness DJ. Neuroimaging-evident lesional pathology associated with REM sleep behavior disorder. *Sleep Med* 2015; 16: 1502-1510.

17. Felix S, Thobois S, Peter-Derex L. Rapid eye movement sleep behaviour disorder symptomatic of a brain stem cavernoma. *J Sleep Res* 2016; 25: 211-215.
18. Flanaghan E, Gavrilova R, Boeve B, et al. Adult onset autosomal dominant leukodystrophy presenting with REM sleep behavior disorder. *Neurology* 2013; 80: 118-120.
19. Tippmann-Peikert M, Boeve BF, Keegan M. REM sleep behavior disorder initiated by acute brainstem multiple sclerosis. *Neurology* 2006; 66: 1277-1279.
20. Schenck CH, Mahowald MW. Injurious sleep behavior disorders (parasomnias) affecting patients on intensive care units. *Intensive Care Med* 1991; 17: 219-224.
21. Enriquez-Marulanda A, Quintana-Pena V, Takeuchi Y, et al. Rapid eye movement sleep behaviour disorder as the first manifestation of multiple sclerosis. *Int J MS Care* 2018; 20: 180-184.
22. Henriques-Filho PSA, Pratesi R. Sleep apnea and REM sleep behavior disorder in patients with Chiari malformations. *Arq Neuropsiquiatr* 2008; 66: 344-349.
23. Henriques-Filho PSA, Pratesia R. Sleep disorder: a possible cause of attention deficit in children and adolescents with Chiari malformation type II. *Arq Neuropsiquiatr* 2009; 67: 29-34.
24. Boeve BF, Silber MH, Ferman TJ, Lucas JA, Parisi JE. Association of REM sleep behavior disorder and neurodegenerative disease may reflect an underlying synucleinopathy. *Mov Disord* 2001; 16: 622-630.
25. Gagnon JF, Petit D, Fantini ML, et al. REM sleep behavior disorder and REM sleep without atonia in probable Alzheimer's disease. *Sleep* 2006; 29: 1321-1325.

26. Anderson KN, Jamieson S, Graham AJ, Shneerson JM. REM sleep behaviour disorder treated with melatonin in a patient with Alzheimer's disease. *Clin Neurol Neurosurg* 2008; 110: 492-495.

27. Postuma RB, Gagnon JF, Vendette M, Fantini ML, Massicotte-Marquez J, Montplaisir J. Quantifying the risk of neurodegenerative disease in idiopathic REM sleep behavior disorder. *Neurology* 2009; 72: 1296-1300.

28. Kim HJ, Im HK, Kim J, et al. Brain atrophy of secondary REM-sleep behavior disorder in neurodegenerative disease. *J Alzheimers Dis* 2016; 52: 1101-1119.

29. Wang P, Wing YK, Xing J, et al. Rapid eye movement sleep behavior disorder in patients with probable Alzheimer's disease. *Aging Clin Exp Res* 2016; 28: 951-957.

30. Youn S, Kim T, Yoon IY, et al. Progression of cognitive impairments in idiopathic REM sleep behaviour disorder. *J Neurol Neurosurg Psychiatry* 2016; 87: 890-896.

31. Abenza Abildua MJ, Miralles Martinez A, Arpa Gutierrez FJ, et al. Conditions associated with REM sleep behaviour disorder: description of a hospital series. Neurologia 2019 34: 159-164.

32. Schenck CH, Garcia-Rill E, Skinner RD, et al. A case of REM sleep behavior disorder with autopsy-confirmed Alzheimer's disease: postmortem brain stem histochemical analyses. *Biol Psychiatry* 1996; 40: 422-425.

33. Fukutake T, Shinotoh H, Nishino H, et al. Homozygous Machado-Joseph disease presenting as REM sleep behaviour disorder and prominent psychiatric symptoms. *Eur J Neurol* 2002; 9: 97-100.

34. Iranzo A, Munoz E, Santamaria J, Vilaseca I, Milà M, Tolosa E. REM sleep behavior disorder and vocal cord palsy in Machado-Joseph disease. *Mov Disord* 2003; 18: 1179-1183.

35. Teive HAG, Arruda WO, Moro A, Moscovich M, Munhoz RP. Differential diagnosis of sporadic adult-onset ataxia: the role of REM sleep behavior disorder. *Parkinsonism Relat Disord* 2015; 21: 640-643.

36. Shindo K, Sato T, Murata H, et al. Spinocerebellar ataxia type 31 associated with REM sleep behaviour disorder: a case report. *BMC Neurol* 2019; 19: 9.

37. Pedroso JL, Braga-Neto P, Felicio AC, et al. Sleep disorders in Machado-Joseph disease: a disease transporter imaging study. *J Neurol Sci* 2013; 324: 90-93.

38. Seyed BH, Rye DB, Singh G. REM sleep behavior disorder and SCA-3 (Machado Joseph disease). *Neurology* 2003; 60: 148

39. Iranzo A, Graus F, Clover L, et al. Rapid eye movement sleep behavior disorder and potassium channel antibody-associated encephalitis. *Ann Neurol* 2006; 59: 178-181.

40. Compta Y, Iranzo A, Santamaria J, Casamitjana R, Graus F. REM sleep behavior disorder and narcoleptic features in anti-Ma2-associated encephalitis. *Sleep* 2007; 30: 767-769.

41. Lin FC, Liu CK, Hsu CY. Rapid-eye-movement sleep behavior disorder secondary to acute aseptic limbic encephalitis. *J Neurol* 2009; 256: 1174-1176.

42. Adams C, McKeon A, Silber MH, Kumar R. Narcolepsy, REM sleep behavior disorder, and supranuclear gaze palsy associated with Ma1 and Ma2 antibodies and tonsillar carcinoma. *Arch Neurol* 2011; 68: 521-524.

43. Dauvilliers Y, Bauer J, Rigau V, et al. Hypothalamic immunopathology in anti-Ma associated diencephalitis with narcolepsy-cataplexy. *JAMA Neurol* 2013; 70: 1305.

44. Vale TC, Fernandes do Prado LB, do Prado GF, Povoas Barsottini OG, Pedroso JL. Rapid eye movement sleep behavior disorder in paraneoplastic cerebellar degeneration: improvement with immunotherapy. *Sleep* 2016; 39: 117-120.

45. Blattner MS, de Bruin GS, Bucelli RC, Day GS. Sleep disturbances are common in patients with autoimmune encephalitis. *J Neurol* 2019; 266: 1007-1015.

46. Gaughan T, Buckley A, Hommer R, et al. Rapid eye movement sleep abnormalities in children with Paediatric Acute-Onset Neuropsychiatric Syndrome (PANS). *J Clin Sleep Med* 2016; 12: 1027-1032.

47. Manni R, Tarzaghi M. REM behavior disorder associated with epileptic seizure. *Neurology* 2005; 64: 883-884.

48. Manni R, Terzaghi M, Zambrelli E. REM sleep behavior and epileptic phenomena: clinical aspects of the comorbidity. *Epilepsia* 2006; 47(suppl 1): 78-81.

49. Manni R, Terzaghi M, Zambrelli E. REM sleep behaviour disorder in elderly subjects with epilepsy: frequency and clinical aspects of the comorbidity. *Epilepsy Res* 2007; 77: 128-133.

50. Arnulf I, Merino-Andreu M, Bloch F, et al. REM sleep behavior disorder and REM sleep without atonia in patients with progressive supranuclear palsy. *Sleep* 2005; 28: 349-354.

51. De Cock VC, Lannuzel A, Verhaeghe S, et al. REM sleep behavior disorder in patients with guadeloupean parkinsonism, a tauopathy. *Sleep* 2007; 30: 1026-1032.

52. Compta Y, Marti MJ, Rey MJ, Ezquerra M. Parkinsonism, dysautonomia, REM behaviour disorder and visual hallucinations mimicking synucleinopathy in a patient with progressive supranuclear palsy. *J Neurol Neurosurg Psychiatry* 2009; 80: 578-579.

53. Sixel-Döring F, Schweitzer M, Mollenhauer B, Trenkwalder C. Polysomnographic findings, video-based sleep analysis and sleep perception in progressive supranuclear palsy. *Sleep Med* 2009; 10: 407-415.

54. Verma A, Anand V, Verma NP. Sleep disorders in chronic traumatic brain injury. *J Clin Sleep Med* 2007; 3: 357-62.

55. Elliott JE, Opel RA, Pleshakov D, et al. Posttraumatic stress disorder increases the odds of REM sleep behaviour disorder and other parasomnias in Veterans with and without comorbid traumatic brain injury. *Sleep* 2019; 43; zsz237.

56. Tribl GG, Bor-Seng-Shu E, Trindale MC, Lucato LT, Teixeira MJ, Barbosa ER. Wilson's disease presenting as rapid eye movement sleep behavior disorder: a possible window to early treatment. *Arq Neuropsiquiatr* 2014; 72: 653-658.

57. Tribl GG, Trindela MC, Bittencourt T, et al. Wilson's disease with and without rapid eye movement sleep behavior disorder compared to healthy matched controls. *Sleep Med* 2016; 17: 179-185.

58. Arnulf I, Nielson J, Lohmann E, et al. Rapid eye movement sleep disturbances in Huntington disease. *Arch Neurol* 2008; 65: 482-488.

59. Ebben MR, Shahbazi M, Lange DJ, Krieger AC. REM behavior disorder associated with familial amyotrophic lateral sclerosis. *Amyotrophic Lateral Scler* 2012; 13: 473-474.

60. Lo Coco D, Puligheddu M, Mattaliano P, et al. REM sleep behaviour and periodic leg movements during sleep in ALS. *Acta Neurol Scand* 2017; 135: 219-224.

61. Kang P, de Bruin GS, Wang LH, et al. Sleep pathology in Creutzfeldt-Jakob Disease. *J Clin Sleep Med* 2016; 12: 1033-1039.

62. Gaig C, Iranzo A, Cajochen C, et al. Characterization of the sleep disorder of anti-IgLON5 disease. *Sleep* 2019; 42: zsz133.

63. Kim H, Yun JY, Choi K-G. Sleep related problems as a nonmotor symptom of dentatorubropallidoluysian atrophy. *J Korean Med Sci* 2018; 33: e130.

64. Puligheddu M, Congiu P, Laccu I, et al. Overlap parasomnia disorder in a case of Creutzfeldt-Jakob Disease. *Sleep Med* 2017; 36: 75-77.

65. Anderson K, Shneerson J, Smith I. Möbius syndrome in associated with the REM sleep behaviour disorder. *J Neurol Neurosurg Psychiatry* 2007; 78: 659-660.

66. Lo Coco D, Cupidi C, Mattaliano A, Baiamonte V, Realmuto S, Cannizzaro E. REM sleep behavior disorder in a patient with frontotemporal dementia. *Neurol Sci* 2012; 33: 371-373.

67. Chokroverty S, Bhat S, Rosen D, Farheen A. REM behavior disorder in myotonic dystrophy type 2. *Neurology* 2012; 78: 2004.

68. Lopez R, Rivier F, Chelly J, et al. Impaired glycinergic transmission in hyperekplexia: a model of parasomnia overlap disorder. *Ann Clin Transl Neurol* 2019; 6: 1900-1904.

69. Trajanovic NN, Voloh I, Shapiro CM, et al. REM sleep behaviour disorder in a child with Tourette's syndrome. *Can J Neurol Sci* 2004; 31: 572-575.

70. Thirumalai SS, Shubin RA, Robinson R. Rapid eye movement sleep behavior disorder in children with autism. *J Child Neurol* 2002; 17: 173-8.
